# Apparent speed of motion concomitant with action alters with delay

**DOI:** 10.1371/journal.pone.0212105

**Published:** 2019-02-15

**Authors:** Yasunaga Monno, Rumi Hisakata, Hirohiko Kaneko

**Affiliations:** 1 Department of Information Processing, Interdisciplinary Graduate School of Science and Engineering, Tokyo Institute of Technology, Yokohama, Kanagawa, Japan; 2 Department of Information and Communications Engineering, School of Engineering, Tokyo Institute of Technology, Yokohama, Kanagawa, Japan; University of Sussex, UNITED KINGDOM

## Abstract

Multiple studies have shown action to affect perception of motion. The speed intended in the generation of a motion by action affects the apparent speed of the motion. However, it was unclear whether action with no intention of speed affects the apparent speed of a motion. In Experiment 1, we investigated the apparent speed of a motion following a key press action. We manipulated the delay from the action to the consequent motion for shifting the timing of efference copy and found the apparent speed decreasing with increases in the delay. This could be because it is known that speed irrelevant action caused expansion of perceived duration of the consequent stimulus and it might have influenced the result in Experiment 1, we investigated the apparent duration of the action consequent static (Ex. 2-1) and motion (Ex. 2-2) stimulus. We found that the apparent duration was not changed with delay. Moreover, the apparent speed and duration had different characteristics on delay. These results were discussed in terms of the sense of agency.

## Introduction

Multiple studies have reported an interaction occurring between action and perception, regardless of modalities or the features of the stimulus: an attenuation of perceived pressure and tickling of one’s own actions [1, 2], consequent sensory effects [3, 4], reduced flash-lag effect with actions [5], the improvement of visual searches for targets related to actions [6], the dependence of estimated distances on the way to reach the target [7] and so on. The interaction between action and perception indicates that the planning and execution of action influence sensory processing [8, 9].

The perception of motion, especially the perception of its direction, relates to action [8, 10]. Wohlschläger [8] found that the perceived direction of ambiguous motion in two frames tended to be congruent with the direction of action, such as the rotation of hand and the press of a key. Mitsumatsu [10] found that in Michotte’s launching stimulus [11], in which two objects approach, intersect, and leave each other and have two interpretations, namely, that they pass through or bounce off one another, the observers tended to perceive the stimulus as moving parallel to the manner of the participant’s hand movement.

The perception of the speed of motion also seems to be related to action. In the previous study, we found that the apparent speed of motion induced by action (we will call such kind of motion “action-consequent motion”) changed with the speed that their participants intended to impart to the stimulus [12]. The participants were able to control the movements of the stimulus, as the stimulus moved in the same direction and speed as their hand. In the condition where the participants were instructed to move the stimulus as quickly as possible, they tended to perceive the resulting stimulus as being faster than it actually was. However, the apparent speed did not change in the conditions where participants were instructed to move the stimulus freely or slower. This indicates that an action affects the apparent speed of the consequent stimulus only when participants have a strong intention related to increased object speed.

However, it is still unclear whether an action irrelevant to the stimulus-motion speed changes the apparent-motion speed; if it does, there would be two possible explanations: either an action affects the apparent-motion speed directly or it affects the apparent-motion speed indirectly via the change in the apparent duration of the motion stimulus because the duration of stimuli following an action (e.g. saccade) was known to appear to be longer in consequent stimuli (chronostasis) [13, 14], resulting in that the estimation of speed will decrease if the motion system calculates the speed as spatial displacement per an unit time. Because speed changes proportionally with duration when the movement distance is physically fixed, the change in apparent speed of a moving stimulus should covary with the apparent duration of that stimulus.

In this study, we investigated the influence of action by manipulating the delay after action before the consequent stimuli because a large delay causes a mismatch between efference copies and upcoming sensory stimuli [1] and decreases the influence of action on the perception of consequent stimuli [3, 15, 16]. Initially, we examined whether and how the apparent speed of the consequent motion changes depending on the delay after the initial action. In Experiment 1, we investigated how the apparent speeds of action-consequent motion changed due to delay after action. We measured the apparent speeds by manipulating the delay of moving stimulus onset from a key press action. As a result when the delay was long, the apparent speed of the moving stimulus was reduced. As noted above, this result may be due to a contraction in apparent duration. Thus, in Experiment 2, we measured the changes in the apparent duration of consequent stimuli with delay. We adopted static stimulus in Experiment 2-1 and motion stimulus in Experiment 2-2. The change of the apparent duration had different characteristics from the apparent speed, suggesting that the effects of action on the perception of speed were independent from time perception.

## Experiment 1

In Experiment 1, we quantitatively investigated whether delays between actions and the onset of stimulus motions changes the apparent stimulus speeds. The participants were asked to compare the apparent speeds of the test stimuli and the comparison stimuli. These stimuli were presented sequentially. The test stimuli were presented with delay and the comparison stimuli without. The ratios of stimulus speeds at the points of subjective equality (PSEs) were calculated as an index.

### Materials and methods

#### Participants

In all, eight students and staff at the Tokyo Institute of Technology, aged between 23 and 33, including two of the authors, participated in the experiment. All the participants had normal or corrected-to-normal vision. All were naive to the purpose of this study, apart from the authors. This experiment was approved by Tokyo Institute of Technology Epidemiological Research Ethics Committee and conducted in accordance with the Code of Ethics in World Medical Association Declaration of Helsinki. Written informed consent was obtained from each observer before the beginning of the experiment.

#### Apparatus and stimuli

**Fig 1:**
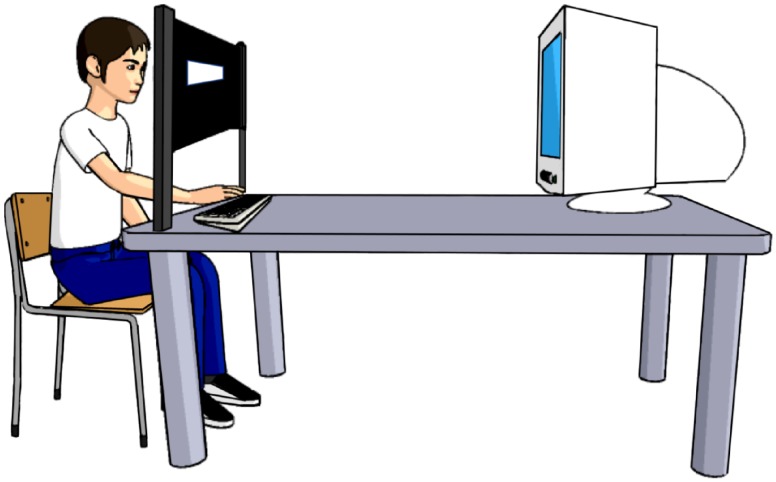
Side view of the experimental apparatus.

Visual stimuli were presented on a 19-inch CRT monitor (SONY GDM-F400, 1024 × 768 pixels, 34.3 × 25.7 cm). The refresh rate was 120 Hz. The viewing distance was approximately 63 cm, giving a visual angle of 30.5° × 23.1°. The participants observed the stimuli through a slit in a partition, making viewing distance and position almost constant throughout the experiment. The height of the slit was manipulated to keep the participants from seeing their hands during the trials (Fig 1). The participants responded to stimuli by pressing keys on a computer keyboard. The experiment was conducted in a dark room.

**Fig 2:**
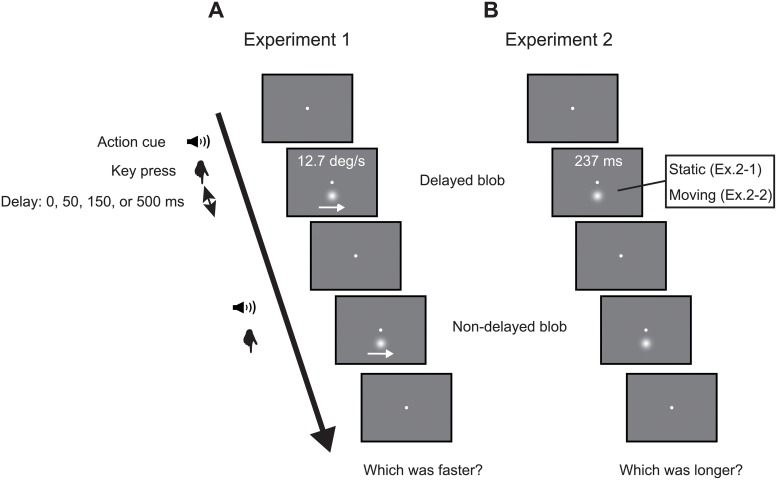
Flows of experimental trials. This shows a trial where the delayed blob was presented first. (A) The participants observed two stimuli presented in sequence and responded in 2IFC to indicate which was faster. (B) The participants indicated which appeared for a longer period.

A white fixation point (90.5 cd/m^2^), with a diameter of 0.15°, was presented at the center of a gray background (15.2 cd/m^2^). Two white moving Gaussian blobs, whose luminance was spatially distributed in Gaussian distribution, with a sigma of 1.04° (90.5 cd/m^2^ on the center), were presented in sequence (Fig 2A). The blobs moved rightward 3°, starting at 1.5° left of the center of the display. The path of motion was 2° below the fixation point. One of the two blobs (the test stimulus) began to move with a delay (0, 50, 150, or 500 ms) after the participants’ key press. The speed was 12.7°/s and the duration of presentation was 237 ms. The other blob (the comparison stimulus) began to move immediately after the key press (no delay), and its speed and, thus, duration of presentation varied, depending on the previous responses (see Procedure section for details).

#### Procedure

We used a two-interval forced-choice (2IFC) method. At the start of the trial, a fixation point was presented. After a random interval of 500-1000 ms after the start of the trial, a tone was presented as an action cue, and the participants pressed the ENTER key. After the key press, a blob (delayed or non-delayed) was presented. After the disappearance of the blob, another tone was similarly presented. The participants pressed the key again and a blob (non-delayed if previous was delayed or delayed if previous was non-delayed) was presented. The participants stated which blob was faster, using the keyboard (Fig 2A), using the 2IFC manner. The order of presentation was random to avoid response biases.

Previous studies [3, 4] have shown that mean delay within an experimental block affects reported stimulus onset. For this reason, in the current study, we adopted two experimental block conditions. In the fixed-delay condition, the presentation delay was constant within each block but varied between blocks. Thus, the mean delay for a given block was different for each delay condition (0, 25, 75, or 250 ms respectively). In the randomized-delay condition, all the delay conditions were randomly presented in a block. Thus, the mean delay was always 87.5 ms.

Each block contained four staircase sequences to determine the speed of the comparison stimulus. In the randomized condition, each delay condition was assigned to a separate sequence. The presented speeds in the four neighboring trials were randomly chosen out of different staircases, preventing any sequence from finishing early. The blocks were complete when all staircases had six response reversals. If one staircase reversed six times, but another did not, the completed staircase restarted with a randomly determined initial speed. Initially, the speed of the non-delayed blob was randomly determined, from 10.16°/s to 15.24°/s and the step size was 0.675°/s. Once the response was reversed, it changed to 0.254°/s. After three times reversals, the step size changed to 0.127°/s, remained until the restart of the sequence. The average number of trials per block was 66.1.

#### Analysis

**Fig 3:**
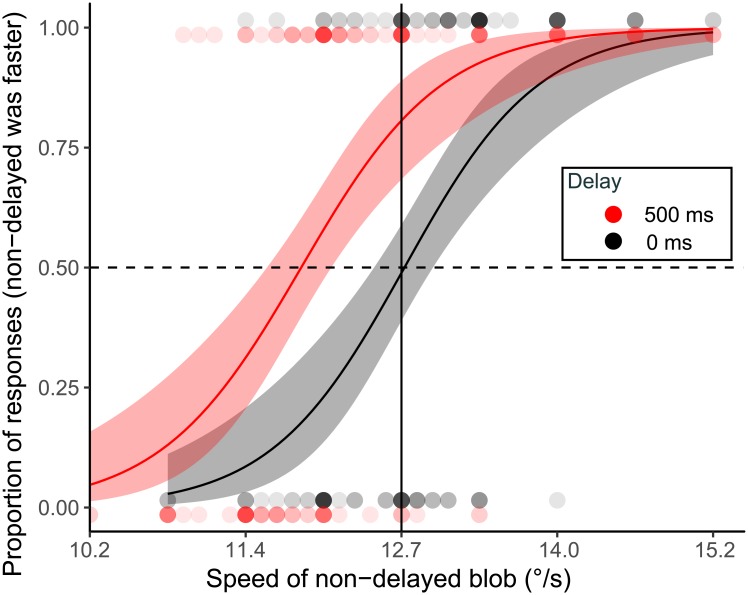
An example of plot of responses and fitted logistic curves. This shows data for 0 and 500 ms delays for the randomized condition. The plotted points indicate responses for the speed of non-delayed blob, and the depth of color indicates the number of responses. Black symbols are data for 0 ms delays and red symbols are for 500 ms delays. Fit curves are depicted and a point of crossing to 0.5 (the broken line) was defined as a PSE. The shaded area indicates the 95% confidence interval. The middle vertical line indicates the speed of delayed blob.

The collected data were analyzed using R, open-source software for statistical computing and graphics. Using the probit analysis, we calculated speeds at the PSEs, and we obtained ratios of the matched speed of comparison (non-delayed) stimulus to that of the test (delayed) stimulus (always 12.7°/s) (Fig 3). We defined this ratio as the apparent speed index (ASI), which represents the effects of delay on apparent speed. Thus, where ASI was less than one, this meant that the participant judged the test (delayed) stimulus slower than the comparison (non-delayed) stimulus. In addition, the 95% confidence interval (CI) of the mean of ASIs in each condition was obtained with bootstrapped ASIs repeated 10,000 times.

### Results and discussion

**Fig 4:**
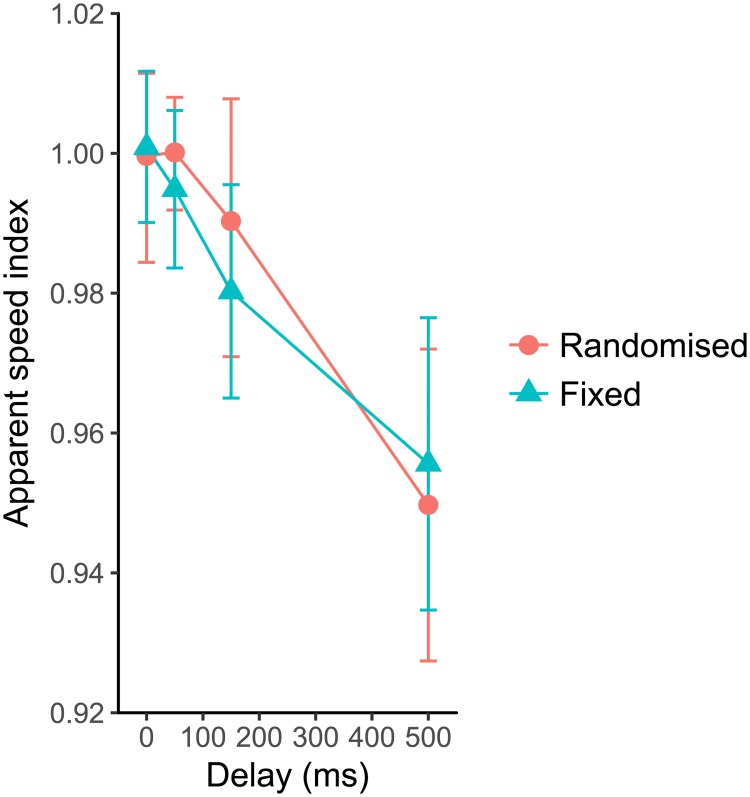
Apparent speed indices as a function of delay. The red circles give the randomized condition and the green triangles give the fixed condition. The error bars depict the 95% CIs of the mean of ASIs obtained with bootstrap method.

Fig 4 shows averaged ASIs across the eight participants, as a function of delay. Circles indicate randomized condition and triangle symbols indicate fixed conditions. Error bars indicate 95% CIs of the means of ASIs obtained with bootstrap method.

The ASIs decreased as delays increased. Furthermore, they did not differ between the randomized and fixed conditions. Repeated-measures ANOVA (delay × block) showed a significant main effect of delay (*F*(3, 21) = 14.9823, *p* < 0.0001, *η*^2^ = 0.3961). On the other hand, there was no significant main effect for block conditions (*F*(1, 7) = 0.1001, *p* = 0.7610, *η*^2^ = 0.0005). The interaction was not significant (*F*(3, 21) = 0.6665, *p* = 0.5819, *η*^2^ = 0.0107). Post-hoc multiple comparison on this main effect with Bonferroni correction indicated significant differences between ASI at 500 ms delay and the others (0 ms, *t*(7) = 4.4689, *p* = 0.0087, *r* = 0.8605; 50 *ms*, *t*(7) = 4.2303, *p* = 0.0117, *r* = 0.8478; 150 *ms*, *t*(7) = 6.0097, *p* = 0.0032, *r* = 0.9152). From the simulated data with bootstrap method, 95% CIs of 0 ms delay conditions did not overlap those of 150 and 500 ms delay conditions. This means that the apparent speed of action-consequent motion decreases as delay increases. Moreover, the absence of interaction between the delay and the block means the averaged delay in the block had no effect on the apparent speed, at least in the given condition.

It was found that the apparent speed of action-consequent motion changed with delays from the action. As mentioned above, this result may be due to changes in the perceived duration of the stimulus rather than changes of the perception of speed itself. Therefore, in Experiment 2 we investigated the apparent durations of the stimuli under the similar conditions to those of Experiment 1.

## Experiment 2

In Experiment 2, we investigated how the apparent duration of action-consequent stimuli change under the same physical delays and durations as in Experiment 1. As it is known that the motion of an object itself affects the perception of time [17, 18], we supposed that the object motion itself would affect the perceived duration. Therefore, we investigated the effects of delay on apparent duration with a static stimulus in Experiment 2-1 and with moving stimulus in Experiment 2-2.

### Materials and methods

#### Participants

In all, 10 students and staff at Tokyo Institute of Technology, including one of the authors, aged between 23 and 39 (Experiment 2-1) and eight students including one of the authors, aged between 22 and 29 (Experiment 2-2) participated in the experiments. All the participants had normal or corrected-to-normal vision. Five participants in Experiment 2-1 also participated in Experiment 1, and two of them including an author participated in Experiment 2-2 (c.f. S2 Table). All except the author were naive to the purpose of this experiment. Written informed consent was obtained from each observer before the experiment.

#### Apparatus, stimuli and procedures

The procedure was similar to that of Experiment 1, except in the following respect. In Experiment 2-1, the Gaussian blob was presented at 2° below the fixation point and did not move. The duration of the (delayed) test stimulus was fixed as 237 ms, and the duration of the comparison (non-delayed) stimulus varied using the staircase method. In one trial, the participants observed the (delayed) test and the (non-delayed) comparison stimuli presented in random order and then responded, using the keyboard, which duration was judged to be longer (Fig 2B). Because we did not find a main effect of block (randomized and fixed conditions) in Experiment 1, we adopted only the randomized condition in this experiment.

In Experiment 2-2, the Gaussian blobs moved rightward during the presentation. The speed of both of the delayed and non-delayed blobs was constant at 12.7°/s. The movement distance of the delayed blob was also fixed at 3° and thus the presenting duration was always 237 ms. On the other hand, the presenting duration of the non-delayed blob varied corresponding to the moving distance. The starting points of them varied randomly by ± 0%–50% of the moving distance for each presentation to prevent participants from judging the duration with disappearing point.

One block consisted of four staircase sequences as in Experiment 1, and the block lasted until all four staircases had seven reversals. Initially, the duration of the non-delayed blob was randomly determined from 189.6 to 284.4 ms, and the step size of the staircase was 23.7 ms. Once the response was reversed, the step size changed to 11.9 ms. After three reversals, it changed to 4.74 ms, remained until being restarted at a random initial duration. The average number of trials for each a block was 76.

#### Analysis

The collected data were analyzed as in Experiment 1 and defined a PSE of duration. We defined the apparent duration index (ADI) to be the ratio of the PSE of the comparison stimulus (non-delayed) to that of the test stimulus (delayed blob). Thus, for ADIs that were more than one, the participant judged the duration of delayed blob longer than the non-delayed blob, where the physical durations were the same. In addition, the 95% CI of the mean of ADIs in each condition was obtained with bootstrapped ADIs repeated 10,000 times as in Experiment 1.

### Results and discussion

**Fig 5:**
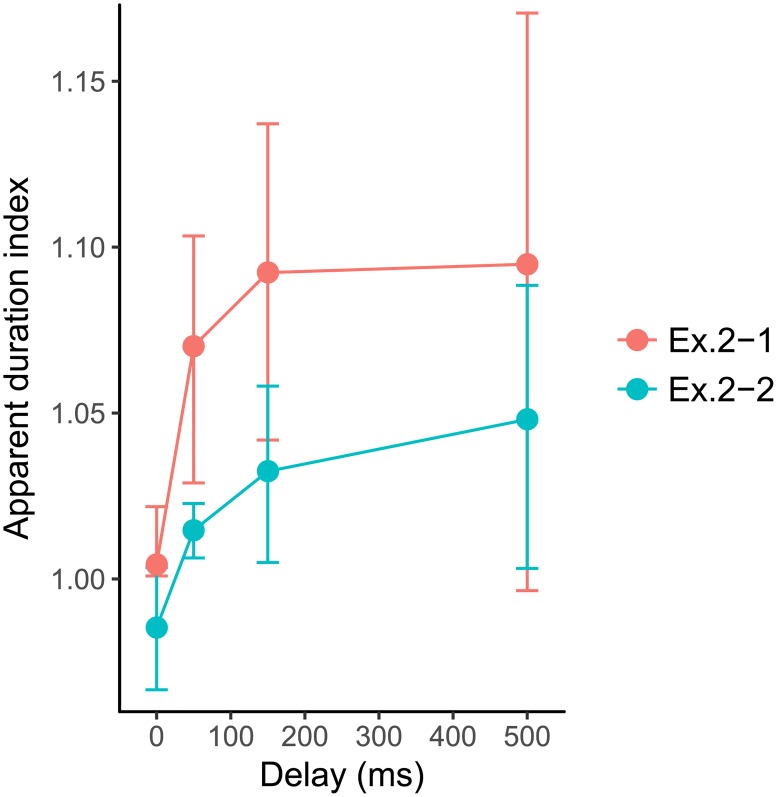
Apparent duration indices as a function of delay. The error bars depict the 95% CIs of the means of ADIs obtained with bootstrap method.

In Experiment 2-1, the data obtained from one of the participants were excluded from further analysis, because the stability of the responses was quite low. The standard error of the estimated PSE was over 10% of the estimated duration in the 0 ms delay condition, indicating that this participant could not correctly judge the difference between the durations in the delayed and non-delayed conditions. Fig 5 shows averaged ADIs of Experiment 2-1 and 2-2 across the remaining nine participants. The error bars indicate confidence intervals of the means of bootstrapped data. Regardless of whether the stimulus was moving, the effects of delay on the apparent duration appeared similar. Even for a 100-ms delay, the apparent duration was larger than that for no delay; there were large individual differences in the duration with a 500-ms delay. Delay seemed to have a larger effect on the static stimulus (Experiment 2-1) than on the moving stimulus (Experiment 2-2). For each experiment, we conducted one-way repeated-measures ANOVA with delay as a factor. In experiment 2-1, the main effect of delay was not significant (*F*(3, 24) = 2.5621, *p* = 0.0785, *η*^2^ = 0.1638). On the other hand, the main effect of delay was significant in Experiment 2-2 (*F*(3, 21) = 3.1591, *p* = 0.0460, *η*^2^ = 0.2640). Post-hoc multiple comparison on this main effect with Bonferroni correction indicated no significant differences. 95% CIs of 50 and 150 ms delay conditions differed from 0 ms delay condition in both Experiment 2-1 and 2-2. We did not find significant differences between 500 ms and other delay conditions, probably due to the large individual differences for the delay condition.

Where the distances for the motion trace were the same, the motion speed and duration were inversely proportional. Although the perception of speed and time do not always follow physical relationships [17], they should be causally related in accordance with the physical relationship, if they are based on a same mechanism.

**Fig 6:**
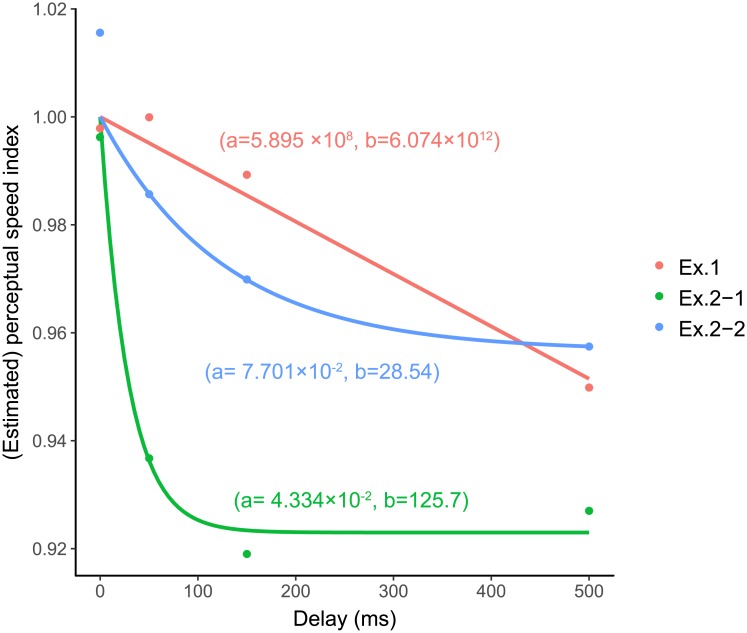
Apparent speed indices in Experiments 1 and estimated apparent speed indices in Experiment 2s. Each curve indicates exponential regression of each experiment. Values beside curves indicate coefficients of each regressions of “ASI = *a* · (*exp*(−delay(ms)/*b*) − 1) + 1”.

Thus, at first, we compared the ASIs of the randomized condition in Experiment 1 and ASIs from Experiment 2-1 and 2-2 (reciprocals of ADIs). Fig 6 shows the ASIs from Experiment 1 and the reciprocals of ADIs from Experiment 2s together. The effect of delay on apparent speed (Exp.1) seemed to be different from the estimated apparent speed from the apparent duration (Exp.2-1, 2-2). For a quantitative comparison, we fitted an exponential function to them.

$$\begin{array}{c} \text{ASI}=a\cdot (exp(-\text{delay}(\text{ms})/b)-1)+1 \end{array}$$

The parameter b determined the curvature, indicating how the delay affected them. In this model, the value of ASI is theoretically 1 at zero-delay. A large value of b indicates that the function is nearly linear and a small value indicates that the function is convex downward. The fitting was successful (Exp. 1: *a* = 5.895 × 10^8^, *b* = 6.074 × 10^12^, adjusted R^2^ = 0.9687, *F*(1,2) = 93.88, *p* = 0.0105, AIC = −29.3268; Exp. 2-1: *a* = 7.701 × 10^-2^, *b* = 28.54, adjusted R^2^ = 0.9853, *F*(1,2) = 202.2, *p* = 0.0049, AIC = −29.1326; Exp. 2-2: *a* = 4.334 × 10^-2^, *b* = 125.7, adjusted R^2^ = 0.9858, *F*(1,2) = 209.2, *p* = 0.0048, AIC = −31.9095).

However, the values of the coefficients were very large for Experiment 1; the regression model might not be suitable for Experiment 1. Thus, we also conducted a linear regression of the model of

$$\begin{array}{c} \text{ASI}=a\cdot \text{delay}(\text{ms})+1 \end{array}$$

and got coefficients (Exp. 1: *a* = 1.000 × 10^−4^, adjusted R^2^ = 0.9687, *F*(1,2) = 93.88, *p* = 0.0105, AIC = −30.0455; Exp. 2-1: *a* = 2.000 × 10^−4^, adjusted R^2^ = −0.04077, *F*(1,2) = 0.8825, *p* = 0.4467, AIC = −10.5393; Exp. 2-2: *a* = 1.000 * 10 − 4, adjusted R^2^ = 0.5211, *F*(1,2) = 4.264, *p* = 0.1749, AIC = −19.7694). For Experiment 1, the AICs of the linear model was smaller than the exponential model and this indicated the linear model was better for the result of Experiment 1 [19]. On the other hand, the exponential model had smaller AICs for Experiments 2-1 and 2-2, which in fact indicates that the exponential model was better for the results of Experiment 2. Therefore, the results of Experiment 1 and 2 had different delay characteristics.

Moreover, we conducted bootstrap resampling and fittings 10,000 times for each experiment and calculated 95% CIs of parameter b in each exponential function. The estimated CIs of parameter b of Experiment 1 did not overlap with those of the other two parameters, whereas the CIs of parameter b in Experiments 2-1 and 2-2 overlapped each other (Exp. 1: CI = [3.665 × 10^12^, 1.102 × 10^13^], median = 5.869 × 10^12^; Exp. 2-1: CI = [1.210, 1.5433 × 10^10^], median = 27.97; Exp. 2-2: CI = [1.450, 2.741 × 10^10^], median = 142.8). This also suggests that the influence of delay differed for Experiment 1 (apparent speed) and Experiment 2 (apparent time).

## General discussion

In this study, we examined the relationship between perception of speed and time of stimuli initiated by an observer’s action. In Experiment 1, we investigated the apparent speed of a moving object whose motion was initiated by the observer’s key press as a function of delay between the action and the onset of stimulus. We found that the apparent speed of the consequent motions decreased as the delay increased. In Experiment 2s, we investigated apparent duration of static and moving objects presented by the observer’s key press as a function of delay. We found that the effects of delay on the apparent duration were not significant on static stimulus and were significant on moving stimulus.

Additionally, we examined whether the effects of delay from action on speed perception were causally related to the effects on perception of duration by comparing the results of Experiments 1 and 2s. We fitted a linear function and an exponential function for the data of each experiment and found that the linear model was better for Experiment 1 and the exponential model was better for Experiment 2s. Moreover, we fitted exponential functions for bootstrapped data of each experiment and found that CIs of a parameter determining curvature of the function in Experiment 1 and in Experiment 2s did not overlap. These results indicated that the influence of action on apparent speed and the influence on apparent duration were based on different mechanisms.

It is to be assumed that the first step in creating a goal of action is not responsible for the effects on speed perception obtained in the present results. This is because, first, the speed of the stimulus presented after the action was almost constant during the experiment, and the participants could not influence the stimulus speed. Second, the task of the action (key press) itself was not related to the speed. Thus, any intention about stimulus speed on the part of the participants during key-press actions was irrelevant. In a previous study, we found that the intention of action was important, because we have shown that the intention related to the speed of stimulus had affected the perceived speed [12], but this result was not valid here. In addition, we consider that the change of perception of speed seen in the current study occurred due to another factor related to the action, not due to other steps contained in the key-press action itself.

Although we believe that an action itself affects the apparent speed of the consequent motion, the result of this study could be influenced by not action itself but perceptual causality between key press action and the stimulus. In this study, we aimed to investigate the effect of an action on speed and duration perception by manipulating the delays between the action and its consequent stimulus. This was based on the fact that large delay mismatches an efference copy and an upcoming signal [1, 20, 21]. However, it is known that delay between events also causes the decrease of perceptual causality between them [22, 23]. We could not isolate the effects of action itself and perceptual causality in the current paradigm because delay could change both of them. However, there are studies reporting that the self-producing action played an important role for establishing the relationship between an action and the consequence [9, 16, 20]. Thus, to deal with this problem, we will need to investigate whether externally caused action, which contains tactile and proprioceptive stimulation but efference copies, affects the apparent speed of the consequent motion and how the effect varies with delay.

One possible factor is the degree of the sense of agency present, which is the sense of causing or generating an event on our own [20, 21, 24]. The sense of agency of the stimulus that was presented immediately after the execution of action should be strong, decreasing with delays from an action to the presentation of the consequent stimulus. The range of delay for the creation of a sense of agency has been reported to be around 150 to 500 ms or less [25, 26]. In Experiment 1, the apparent speed for the stimulus at 500 ms of delay, which is about the same as the upper limit of delay for the sense of agency, was slower than that for the stimulus with a shorter delay. It has been found that the sense of agency affects the visual processing, for example the reaction time for the stimulus presented neighboring the object regarded as the agent [9]. However, no conclusive evidence is available at present, because we did not measure the participants’ sense of agency in the current study. In future studies, the effects of the sense of agency on apparent speed must be investigated by comparing both directly.

It was possible that the effects of delay on apparent speed and duration were not caused by the change in perception but by the change in the criteria of judgment because we adopted the 2IFC paradigm in this study [27, 28]. We did not adopt the 2AFC paradigm for our experiments because if the standard and comparison stimuli were presented simultaneously, participants could report the speed difference depending on relative position or temporal difference of standard and comparison stimuli (not depending on speed difference itself). In this study, we primarily desire to report whether a delay between key press action and its consequent stimulus affected the “appearance of the consequent motion.” For this reason, we could not distinguish the effect of action on perception and judgment in the scope of this study. In further studies, we will try to investigate the response, decision, and perception biases respectively with 3- or 4-IFC paradigm.

The results of this study indicate that actions adjacent to stimulus onset can change the apparent speeds even without the intention of modifying stimulus speed. In addition, the change in the perception of the speed of motion initiated by action and the change in the perception of time due to action are based on different mechanisms. However, because the characteristics of the perception of speed can be changed in different speed ranges [29, 30], it is necessary to investigate how the perception of speed change in action for a broader range of speeds to fully understand the effects of action on the perception of speed.

## Supporting information

S1 Table Data for all trials. (XLSX)

S2 Table Perceptual indices for all participants. (XLSX)

S3 Table Calculation of AICs. (XLSX)
